# Prescription of Antibiotic Prophylaxis for Dental Implant Surgery in Healthy Patients: A Systematic Review of Survey-Based Studies

**DOI:** 10.3389/fphar.2020.588333

**Published:** 2021-02-10

**Authors:** Juan Carlos Bernabeu-Mira, Miguel Peñarrocha-Diago, David Peñarrocha-Oltra

**Affiliations:** Department of Stomatology, Faculty of Medicine and Dentistry, University of Valencia, Valencia, Spain

**Keywords:** antibiotic, prophylaxis, survey, dental implant, systematic review

## Abstract

**Background:** Systemic antibiotic prophylaxis is frequently prescribed by dentists performing dental implant surgery to avoid premature implant failure and postoperative infections. The scientific literature suggests that a single preoperative dose suffices to reduce the risk of early dental implant failure in healthy patients.

**Material and Methods:** A systematic review was made based on an electronic literature search in the PubMed-Medline, Embase, Web of Science, Scopus and Open Gray databases. The review addressed the question: “which antibiotic prophylaxis regimens are being used in dental implant surgery in healthy patients according to survey-based studies?” The identification, screening, eligibility and inclusion phases were conducted according to the PRISMA statement by two independent reviewers. The following data were collected: country, number of surveyed dentists, number of dentists who responded (n), response rate, routine prescription of antibiotic prophylactic treatment (yes, no, or conditioned prescription), prescription regimen (preoperative, perioperative or postoperative) and antibiotic choice (first and second choice). Cohen’s kappa coefficient (k) evaluated the level of agreement between the two reviewers. The analysis of risk of bias was performed follow the Joanna Briggs Institute checklist for observational studies. A descriptive statistical analysis was performed to calculate total target sample, sample size and total mean.

**Results:** A total of 159 articles were identified, of which 12 were included in the analysis. Two thousand and seventy-seven dentists from nine different countries on three continents were surveyed. The median response rate was low and disparate between studies. About three-quarters of the surveyed dentists claimed to routinely prescribe systemic antibiotic prophylaxis for dental implant surgery. The prescription regimen was perioperative, postoperative and preoperative, in decreasing order of frequency. The most frequent first choice drug was amoxicillin, with amoxicillin-clavulanic acid as second choice.

**Conclusions:** A majority of dentists from different countries do not prescribe systemic antibiotic prophylaxis for dental implant surgery following the available scientific evidence and could be overprescribing. Efforts are needed by dental educators and professionals to reduce the gap between the use of antibiotic prophylaxis for dental implant surgery as supported by the scientific evidence and what is being done by clinicians in actual practice.

## Introduction

According to the World Health Organization (WHO), the overprescription of antibiotic drugs and the derived antimicrobial resistances constitute one of the most pressing public health problems in the world today (World Health Organization, 2012). In turn, antibiotic use has been associated to side effects in the form of secondary infection, toxicity, allergic reaction, rush, nausea and diarrhea (Granowitz and Brown, 2008).

According to a systematic review of qualitative studies by Teixeira Rodrigues et al. (2013), the most influential variables in physician antibiotic prescribing behavior were complacency with patient expectations, and fear of possible future complications or of losing the patient. Certain healthcare system-related factors such as time pressure considerations and policies or guidelines may also exert an influence (Teixeira Rodrigues et al., 2013).

Dental implants are currently a routine treatment, having demonstrated their long-term success (Simonis et al., 2010); as a result, the number of dental implant treatments has increased over time (Ng et al., 2011).

Early dental implant failure occurs approximately in 2% of all cases and is characterized by a lack of osseointegration of the implant (Troiano et al., 2018). It has been associated to intraoperative (lack of dental implant stability, implant contamination or trauma during surgery (Sakka et al., 2012)) and postoperative factors (micromovements exceeding 150 µm (Frost, 2004)). Bacterial colonization of the peri-implant site has also been cited (Esposito et al., 1998). The bacterial spectrum associated to early dental implant failure is dominated by streptococci, anaerobic grampositive cocci and anaerobic gramnegative species (Mombelli et al., 1987).

Systemic antibiotic prophylaxis has been used to reduce the risk of early implant failure and local infections around dental implants (Dent et al., 1997; Laskin et al., 2000). Such preventive measures are currently the subject of debate, however.

Recent studies have concluded that the administration of systemic antibiotics does not significantly reduce early implant failure in simple implant surgeries (defined as surgeries without broad flaps or simultaneous bone regeneration) in healthy patients (Mazzocchi et al., 2007; Lund et al., 2015; Romandini et al., 2019). Moreover, local infections after dental implant placement do not benefit from the administration of systemic antibiotics (Khouly et al., 2019).

However, several systematic reviews and meta-analyses (Esposito et al., 2013; Rodríguez Sánchez et al., 2018; Romandini et al., 2019) have proposed the use of a single preoperative antibiotic dose of amoxicillin 1 h before dental implant surgery. This strategy may suffice to reduce the early dental implant failure rate to 2% (Lund et al., 2015). Perioperative and exclusive postoperative antibiotic prophylaxis have yielded equivalent outcomes when compared to exclusive preoperative single-dose prophylaxis in terms of early dental implant failure (Esposito et al., 2013; Rodríguez Sánchez et al., 2018; Romandini et al., 2019). Accordingly, perioperative and exclusively postoperative regimens would imply antibiotic overprescription.

The first and most important step in designing effective interventions to improve prescription practice is to identify and understand antibiotic prescription and its modulating factors in dental implant surgery (Livermore, 2005). The present systematic review was carried out to investigate current trends in antibiotic prescription behavior among dentists of different countries performing dental implant surgery according to survey-based studies.

## Material and Methods

We conducted a systematic review of the prescription of antibiotic prophylaxis for dental implant surgery as evidenced by survey-based studies according to the PRISMA-P (Preferred Reporting Items for Systematic review and Meta-Analysis protocols) statement (Moher et al., 2015).

### Identification Phase and Search Strategy

This systematic review was based on the following PIOS question: “which antibiotic prophylaxis regimens (O) are being used in dental implant surgery (I) in healthy patients (P) according to survey-based studies (S)?” The search strategy was based on the following keywords:

• Patient (P): healthy patients
• Intervention (I): dental implant surgery
• Outcome (O): prophylaxis, prescription, habit, use or attitude
• Study design (S): survey, questionnaire

The identification phase based on the search strategy in several databases was updated on 10 May 2020. For the PubMed-Medline search we used the medical subject heading (MeSH) terms (and their entry terms) and non-MeSH terms. The Embase search in turn was based on Emtree terms and their synonyms, and non-Emtree terms. The Web of Science and Scopus databases were also consulted. A search of the gray literature was also performed in Open Gray to include articles published in non-indexed journals or to retrieve a larger number of studies. Furthermore, a manual search was made of the references of the articles retrieved by the previous search strategies. The search strategy corresponding to each database is indicated in Table 1.

**TABLE 1:** Search strategies to the PubMed-Medline, Embase, Web of Science, Scopus and Open Gray databases.

PubMed-Medline	(((dental implant) and (antibiotic)) and (prescription OR prophylaxis OR use OR habit OR attitude)) and (survey OR questionnaire OR questionary)
Embase	dental and implant and antibiotic and (prescription OR prophylaxis OR use OR habit OR attitude) and (survey OR questionnaire OR questionary)
Web of Science	ALL=(dental implant) and ALL=(antibiotic) and ALL=(prescription OR prophylaxis OR use OR habit OR attitude) and ALL=(survey OR questionnaire OR questionary)
Scopus	ALL (( dental and implant) and (antibiotic) and (prescription OR prophylaxis OR use OR habit OR attitude) and (survey OR questionnaire OR questionary))
Open Gray	Antibiotic dental implant

### Screening and Eligibility Phases

The titles and abstracts were read in the screening phase to eliminate articles unrelated to the study objective and duplicate articles. Full-text reading was subsequently made in the eligibility phase to select studies according to the inclusion/exclusion criteria. We included studies in which antibiotic prophylaxis in dental implant surgery was the reason for prescription, without language or date of publication restrictions. Experimental studies or database research studies were excluded, as were studies involving pregraduate dental students, articles lacking information about the prescription regimens (no distinction between exclusively preoperative, exclusively postoperative or perioperative prescription), and studies lacking information about the antibiotic of choice.

In the case of studies providing information on different dental procedures, only antibiotic prescription referred to dental implant surgery was considered. In addition, if the type of implant surgery was detailed (flap elevation, bone site condition or number of dental implants), data were recorded referred to antibiotic prescription for single implants at mature bone sites with flap elevation. In survey-based studies involving some type of training or educational intervention, the data prior to such intervention were collected.

The screening and eligibility phases were processed in duplicate by two independent reviewers (JCBM and DPO). In the case of disagreement between the reviewers in any phase, a third reviewer (MPD) was consulted. Cohen’s kappa coefficient (k) was used to assess the level of agreement between JCBM and DPO.

### Data Collection

The following variables were collected for each included article: author, year of publication, country, target sample, number of dentists who respond (N), response rate (RR), routine prescription of antibiotic prophylaxis (yes, no or conditioned prescription), prescription modulating factors, prescription regimen (preoperative, postoperative or perioperative) and antibiotic of choice (first and second choice drug).

### Assessment of Risk of Bias

A critical appraisal tool for use in systematic reviews addressing questions of prevalence derived from the Joanna Briggs Institute checklist (Munn et al., 2014) was used to evaluate the methodological quality of the included studies. The checklist comprised 10 items, each being scored as “Yes,” “Unclear,” “No” or “Not applicable.” High risk of bias was considered when a study complied with four items, while moderate risk of bias was considered for five to seven items and low risk of bias for 8–10 items.

### Statistical Analysis

A descriptive statistical analysis was performed using the SPSS version 26.0 statistical package, with calculation of the total target sample, sample size and total mean.

## Results

**FIGURE 1:**
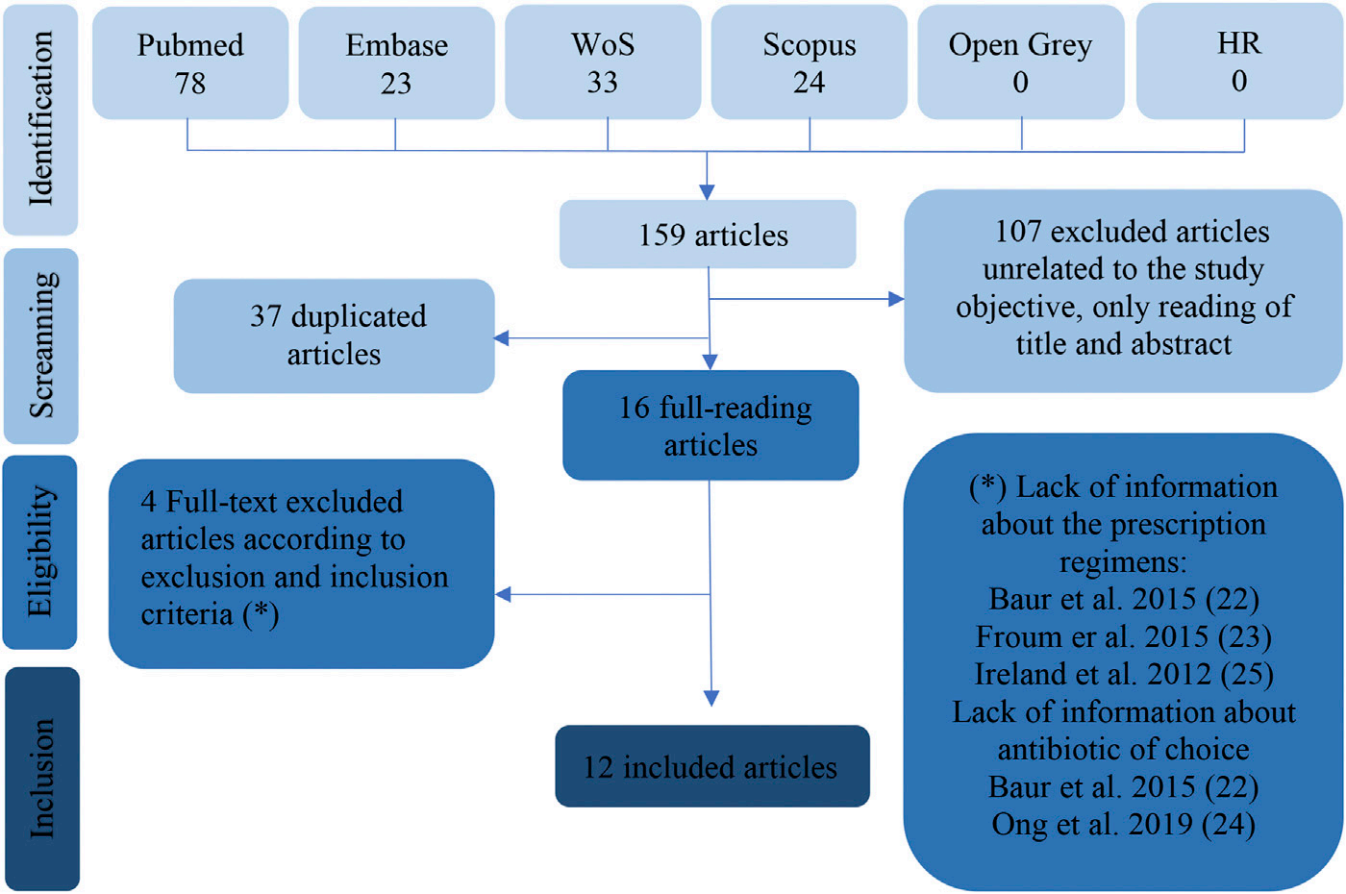
Flowchart corresponding to the selection process of the retrieved articles.

The present systematic review was performed according to the PRISMA flow diagram (Figure 1). A total of 159 search results were filtered (PubMed-Medline: 78 articles, Embase: 23 articles, Web of Science: 34 articles, Scopus: 24 articles, Open Gray: 0 articles and 0 manual search articles). After eliminating duplicate articles and articles unrelated to the study objective, a total of 16 articles were seen to meet the selection criteria. Finally, 12 full-text evaluated articles were included in the systematic review. Cohen’s kappa coefficient (k) was 1. Discrepancy in the screening and eligibility phase was null between JCBM and DPO.

The results of the included studies are summarized in Table 2.

**TABLE 2:** Extracted results of the included studies regarding the prescription of antibiotic prophylaxis in dental implant surgery.

Author/year	Country	TS	*N*	RR (%)	Antibiotic prophylaxis (*N*)			Prescription regimen (*N*)			Antibiotic of choice	
					Yes	Mod.	No	Pre	Peri	Post	First choice	Second choice
AbuKaraky et al., 2011	Jordan	250	172	70.4	140	—	32	20	40	80	Amox. + clav.	Amox.
Marín et al., 2012	Chile	—	33	—	10	14	12	16	8	0	Amox.	Doxici.
Datta et al., 2014	India	350	332	94.8	284	—	48	7	179	98	Penicill.	Amox. + clav.
Deeb et al., 2015	United States	1,436	217	15	192	—	25	40	72	80	Amox.	Penicill.
Khalil et al., 2015	Sweden	120	90	75	68	—	22	15	20	33	Phenoxy Metilpenicillin	Amox.
Al-Kattan and Al-Shibani, 2019	Saudi Arabia	400	109	27.25	65	44	0	14	22	73	Amox. + clav.	Amox.
El-Kholey et al., 2018	Saudi Arabia	133	133	100	133	—	0	78	55	0	Amox.	Amox. +clav.
Camps-Font et al., 2018	Spain	1,227	247	20.1	211	—	36	17	94	100	Amox.	Amox. +clav.
Arteagoitia et al., 2018	Spain	989	233	23.56	207	22	4	13	179	35	Amox.	Amox. +clav.
Camacho-Alonso et al., 2019	Spain	210	200	95.24	94	—	106	14	30	50	Amox. + clav.	Amox.
Rodríguez Sánchez et al., 2019b	Italy	400	160	40	134	25	1	29	116	14	Amox. + clav.	Amox.
Rodríguez Sánchez et al., 2019a	Netherlands	902	151	24.9	66	80	5	47	83	12	Amox.	Amox. +clav.
Total	—	6.417	2.077	31.9	1.604	185	291	310	898	575	—	—

TS, target sample; N, number of samples; RR (%), response rate; Yes, routine prescription of antibiotic prophylaxis; Mod, Prescription modulated by factors; No, no prescription of antibiotic prophylaxis; Pre, exclusively preoperative prescription; Peri, perioperative prescription; Post, exclusively postoperative prescription; Amox., amoxicillin; Amox.+clav, amoxicillin and clavulanic acid; Penicill., penicillin; Doxici., doxycycline.

The target sample was 6,417 dentists, though one study (Marín et al., 2012) failed to report the number. The median RR was 31.9%, i.e., 2,077 dentists answered the survey. The RR was very disparate (15–100%) between studies (Table 2).

The surveyed dentists were from nine different countries on three continents. Six studies corresponded to four countries in Europe: Sweden (Khalil et al., 2015), Spain (Arteagoitia et al., 2018; Camps-Font et al., 2018; Camacho-Alonso et al., 2019) Italy (Rodríguez Sánchez et al., 2019b) and The Netherlands (Rodríguez Sánchez et al., 2019a). Four studies corresponded to three countries in the Middle East and Asia: Jordan (Abukaraky et al., 2011), India (Datta et al., 2014) and Saudi Arabia (El-Kholey et al., 2018; Al-Kattan and Al-Shibani, 2019). Lastly, two studies corresponded to two countries in America: the United States (Deeb et al., 2015) and Chile (Marín et al., 2012) (Table 2).

A total of 77.1% of the surveyed dentists claimed to routinely prescribe antibiotic prophylaxis in healthy patients undergoing dental implant placement. In turn, 8.9% only prescribed antibiotics depending on prescription modulating factors. Fourteen percent claimed to not prescribe antibiotic prophylaxis in any situation (Table 2).

Different prescription modulating factors were recorded in different studies: three clinical factors corresponding to patient conditions, five clinical factors regarding the surgical procedure and eight non-clinical factors. As clinical modulating factors corresponding to the patient conditions, we found past periodontal disease (Arteagoitia et al., 2018; Rodríguez Sánchez et al., 2019a; Rodríguez Sánchez et al., 2019b), smoking (Arteagoitia et al., 2018; Rodríguez Sánchez et al., 2019a; Rodríguez Sánchez et al., 2019b) and heart disease requiring antibiotic prophylaxis (Arteagoitia et al., 2018; Rodríguez Sánchez et al., 2019a; Rodríguez Sánchez et al., 2019b) to influence antibiotic prescription. As clinical modulating factors corresponding to the surgical procedure, we recorded immediate implant placement (Marín et al., 2012; Rodríguez Sánchez et al., 2019a; Rodríguez Sánchez et al., 2019b), bone grafting (Marín et al., 2012; Arteagoitia et al., 2018; Rodríguez Sánchez et al., 2019a; Rodríguez Sánchez et al., 2019b), preoperative implant site infection (Arteagoitia et al., 2018; Rodríguez Sánchez et al., 2019a; Rodríguez Sánchez et al., 2019b), sinus membrane perforation (Arteagoitia et al., 2018; Rodríguez Sánchez et al., 2019a Rodríguez Sánchez et al., 2019b) and simultaneous multiple dental implant placement (Arteagoitia et al., 2018; Rodríguez Sánchez et al., 2019a; Rodríguez Sánchez et al., 2019b). The reported non-clinical modulating factors were (Abukaraky et al., 2011) patient preference, reading scientific publications, knowledge gained during undergraduate or postgraduate training, attending courses and lectures, availability in the nearby pharmacy, advertisement, cost of the antibiotic, recommendations by other colleagues, drug effectiveness, and previous experience with the drug.

**FIGURE 2:**
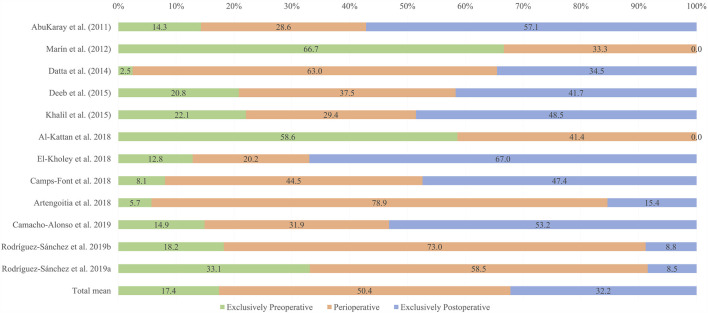
Distribution of the prescription of antibiotic prophylaxis according to author/year. The total mean is also shown.

The distribution of prescription regimens proved heterogeneous both within and between studies. Perioperative prescription was the most frequent regimen (50.4%), followed by exclusive postoperative prescription (32.2%) and finally exclusive preoperative prescription (17.4%). The prescription regimens were seen to vary considerably even between studies from one same country (Figure 2).

Regarding the antibiotic of choice (Table 2), amoxicillin was the most common antibiotic of first choice (Marín et al., 2012; Deeb et al., 2015; Arteagoitia et al., 2018; Camps-Font et al., 2018; El-Kholey et al., 2018; Rodríguez Sánchez et al., 2019), followed by amoxicillin-clavulanic acid (Abukaraky et al., 2011; Al-Kattan and Al-Shibani, 2019; Camacho-Alonso et al., 2019; Rodríguez Sánchez et al., 2019). For the majority of dentists who prescribed amoxicillin as the antibiotic of first choice, amoxicillin-clavulanic acid was the second election, and vice versa. Other antibiotics reported as the most frequent first choice were penicillin (Datta et al., 2014) and phenoxymethylpenicillin (Khalil et al., 2015), with penicillin (Deeb et al., 2015) and doxycycline as the most frequent second choice (Marín et al., 2012).

Regarding the analysis of risk of bias (Table 3), one of the studies exhibited high risk of bias (Marín et al., 2012) while the other studies exhibited moderate risk of bias (Abukaraky et al., 2011; Datta et al., 2014; Deeb et al., 2015; Khalil et al., 2015; Arteagoitia et al., 2018; Camps-Font et al., 2018; El-Kholey et al., 2018; Al-Kattan and Al-Shibani, 2019; Camacho-Alonso et al., 2019; Rodríguez Sánchez et al., 2019; Rodríguez Sánchez et al., 2019). No study was at low risk of bias.

**TABLE 3:** Summary of the risk of bias on the cross-sectional studies included in the systematic review. “Yes” (green); “Unclear” (yellow); “No” (red).

## Discussion

The main objective of the present systematic review was to investigate current trends in antibiotic prescription behavior among dentists of different countries performing dental implant surgery, according to survey-based studies.

The RR varied greatly between studies (in a range of 15–100%), and even within the same country. This could be due to the different sampling methods used. Although the surveys were online in all the studies except one (Datta et al., 2014), the way of getting in touch with the surveyed dentists differed. Studies limited to sending e-mails through affiliation to scientific societies achieved lower RRs (Deeb et al., 2015; Arteagoitia et al., 2018; Camps-Font et al., 2018; Al-Kattan and Al-Shibani, 2019; Rodríguez Sánchez et al., 2019a; Rodríguez Sánchez et al., 2019b). However, several supplementary methods increased the RR, such as telephone calls (Khalil et al., 2015; Camacho-Alonso et al., 2019), calculation of a representative sample size, and focusing on personal approaches during conferences and academic meetings (Datta et al., 2014; El-Kholey et al., 2018; Camacho-Alonso et al., 2019). The studies that only e-mailed dentists compensated the lower RR with a larger target sample.

Most of the surveyed dentists claimed to routinely prescribe systemic antibiotic prophylaxis. Several studies discerned between routine and conditioned prescription depending on the medical conditions of the patient and the complexity of the intended procedure (Marín et al., 2012; Arteagoitia et al., 2018; Al-Kattan and Al-Shibani, 2019; Rodríguez Sánchez et al., 2019a; Rodríguez Sánchez et al., 2019b). Regarding medical conditions, patients with a history of periodontal disease (Rodríguez Sánchez et al., 2019a) and smokers (Sgolastra et al., 2015) exhibited a greater early implant failure rate. According to some of the included studies (Arteagoitia et al., 2018; Rodríguez Sánchez et al., 2019a; Rodríguez Sánchez et al., 2019b), this fact induced the dentist to prescribe antibiotic prophylaxis more often. Patients with heart disease may require antibiotic prophylaxis to prevent infective endocarditis, according to some clinical guides (Manzano et al., 2016). With respect to the complexity of the procedure, antibiotic prescription has resulted in lower dental implant failure rates in immediate implant placement (Habib et al., 2015). The use of preoperative single-dose antibiotic prophylaxis seems to have the same effect as 3 days of postoperative treatment for bone grafting (Cosyn et al., 2019), though the scientific evidence is limited and very recent. The use of antibiotics at perioperative infective sites is subject to debate (Payer et al., 2020), and few published data are available on sinus membrane perforation or multiple simultaneous implant placement.

According to the scientific literature, a single preoperative antibiotic dose of amoxicillin 1 h before surgery could be useful to reduce early dental implant failure. Perioperative and exclusive postoperative antibiotic prophylaxis have demonstrated results equivalent to exclusive single-dose preoperative prophylaxis in relation to early implant failure (Esposito et al., 2013). Thus, the observed high percentages of perioperative (50.4%) and exclusive postoperative prophylaxis (32.2%) imply an overprescription of antibiotics. The few dentists who responded that they exclusively prescribed antibiotic prophylaxis on a preoperative basis could be prescribing in concordance with the current scientific evidence (Esposito et al., 2013). However, the 14% of dentists who exclusively prescribed preoperative antibiotic treatment described different regimens: 2 days before, 1 day before, 12 h before, 8 h before, 1 h before, 30 min before, and immediately before surgery (Deeb et al., 2015; Arteagoitia et al., 2018; Camps-Font et al., 2018; Camps-Font et al., 2018; El-Kholey et al., 2018; Rodríguez Sánchez et al., 2019; Rodríguez Sánchez et al., 2019). Some of these antibiotic prescription regimens also could constitute overprescription practice. As an example, exclusive preoperative antibiotic prescription was the most frequently used regimen in only one study (Marín et al., 2012). However, this article did not describe the selection criteria of the surveyed dentists, and the sample size was the smallest of all the studies, with 33 included dentists.

In all the included studies, the first choice antibiotic corresponded to the penicillin family. The most frequently prescribed first choice antibiotic was amoxicillin (Marín et al., 2012; Deeb et al., 2015; Arteagoitia et al., 2018; Camps-Font et al., 2018; El-Kholey et al., 2018; Rodríguez Sánchez et al., 2019a), followed by amoxicillin plus clavulanic acid (Abukaraky et al., 2011; Al-Kattan and Al-Shibani, 2019; Camacho-Alonso et al., 2019; Rodríguez Sánchez et al., 2019b). The reason for choosing amoxicillin as the antibiotic of choice could be its established evidence in preventing dental implant failure (Chrcanovic et al., 2015). Other reasons are better compliance, good absorption, good bioavailability and a broader bactericidal effect upon the oral microflora (Resnik and Misch, 2008; Kashani et al., 2019). The use of amoxicillin plus clavulanic acid—representing the first choice in several studies (Abukaraky et al., 2011; Al-Kattan and Al-Shibani, 2019; Camacho-Alonso et al., 2019; Rodríguez Sánchez et al., 2019b)—did not provide benefits compared to amoxicillin alone (Aravena et al., 2018). The data on antibiotic dosage were not included in the analysis, because the reviewed studies failed to yield such information in a uniform manner.

The discrepancy between the prescription of antibiotic prophylaxis for dental implant surgery in healthy patients according to the survey-based studies and optimal prescription as suggested by the available evidence could be contributing to the development of antimicrobial resistances (World Health Organization, 2012), and may have negative effects upon patient health (secondary infection, toxicity, allergic reaction, rash, nausea and diarrhea) (Granowitz and Brown, 2008). Educational programs and clinical guidelines should be promoted to improve the use of antibiotic prophylaxis in dental implant surgery. According to Khalil et al. (2015), a strategic program against antibiotic resistance produced a significant difference in terms of the reduction and optimization of antibiotic prophylaxis in dental implant surgery.

In the present systematic review, different types of surveys were used to ask the dentists about their preferences regarding the prescription of antibiotic prophylaxis in dental implant surgery. Two main types of surveys were identified, whose original designs corresponded to Abukaraky et al. (2011) and Deeb et al. (2015). In this regard, we would like to offer some recommendations for future survey-based studies on this topic: compilation of the RR of the target sample; indication of the type of dental implant surgery (flapless or flap approach; simultaneous bone regeneration; single, multiple, immediate or delayed implant placement); restriction of selection (for example, those dentists who answer that they do not prescribe antibiotic prophylaxis should not complete the following sections of the survey); differentiation between routine and conditioned prescription; and differentiation within each prescription regimen of how the dentists prescribe (for example, whether exclusive preoperative prescription began 1 day or 1 h, etc., before surgery). A problem was detected in two studies with the same survey design and without restriction of selection (Arteagoitia et al., 2018; Rodríguez Sánchez et al., 2019b): the number of dentists adding up the prophylactic antibiotic regimen (pre-, peri- or postoperative) outnumbered the dentists who claimed to routinely and occasionally prescribe antibiotic prophylaxis.

Based on the findings of the present systematic review, incorrect and unjustified antibiotic prescription practices in dental implant surgery in healthy patients are observed. The publication of specialized clinical guidelines and continuous and focused training for prescribers are needed. Defective antibiotic prescription and the indiscriminate use of such drugs can produce serious bacterial resistance problems.

As a limitation of the present systematic review, mention must be made of the fact that the number of countries and professionals evaluated in relation to the prescription of antibiotic prophylaxis was limited. As a strength, however, the study evidences that understanding of the use and prescription of antibiotic prophylaxis for implant surgery among dentists should be strengthened. The results of our systematic review were based on studies characterized by moderate and high risk of bias, so the results should be interpreted with caution.

As recommendations, the calculation and selection of representative samples, and the taking of confounding factors into account, may improve the quality of future studies. Moreover, scientific associations could develop a common and proven survey for future studies based on the best available questionnaires and possible improvements.

## Conclusion

According to cross-sectional survey-based studies, a majority of dentists from different countries do not prescribe systemic antibiotic prophylaxis for dental implant surgery following the available scientific evidence and could be overprescribing. Efforts are needed by dental educators and professionals to reduce the gap between the use of antibiotic prophylaxis for dental implant surgery as supported by the scientific evidence and what is being done by clinicians in actual practice.
